# Genetic diversity and population structure of *Physella acuta* (Gastropoda: Physidae) in Thailand using mitochondrial gene markers: COI and 16S rDNA

**DOI:** 10.1038/s41598-024-64184-4

**Published:** 2024-06-07

**Authors:** Abdulhakam Dumidae, Jiranun Ardpairin, Supawan Pansri, Chanatinat Homkaew, Mayura Nichitcharoen, Aunchalee Thanwisai, Apichat Vitta

**Affiliations:** 1 Department of Microbiology and Parasitology, Faculty of Medical Science, Naresuan University, Phitsanulok, 65000 Thailand; 2 Centre of Excellence in Medical Biotechnology (CEMB), Faculty of Medical Science, Naresuan University, Phitsanulok, 65000 Thailand; 3 Center of Excellence for Biodiversity, Faculty of Sciences, Naresuan University, Phitsanulok, 65000 Thailand

**Keywords:** *Physella acuta*, Invasions, Genetic diversity, Gene flow, Thailand, Genetics, Zoology

## Abstract

*Physella acuta* is a freshwater snail native to North America*.* Understanding the phylogeography and genetic structure of *P. acuta* will help elucidate its evolution. In this study, we used mitochondrial (*COI* and 16S rDNA) and nuclear (ITS1) markers to identify the species and examine its genetic diversity, population structure, and demographic history of *P. acuta* in Thailand. Phylogenetic and network analyses of *P. acuta* in Thailand pertained to clade A, which exhibits a global distribution. Analysis of the genetic structure of the population revealed that the majority of pairwise comparisons showed no genetic dissimilarity. An isolation-by-distance test indicates no significant correlation between genetic and geographical distances among *P. acuta* populations, suggesting that gene flow is not restricted by distance. Demographic history and haplotype network analyses suggest a population expansion of *P. acuta*, as evidenced by the star-like structure detected in the median-joining network. Based on these results, we concluded that *P. acuta* in Thailand showed gene flow and recent population expansion. Our findings provide fundamental insights into the genetic variation of *P. acuta* in Thailand.

*Physella acuta* (Draparnaud, 1805) (syn. *Physa acuta*) is a freshwater snail from the Physidae family. This snail is well known for its remarkable ability to invade new habitats. They are classified as freshwater pulmonate snails^1,2^. *Physella acuta* is an invasive snail native to North America^1^. *Physella acuta* exhibits remarkable expansiveness owing to its capacity to disperse through various vectors, broad ecological tolerance, swift adaptation to novel habitats, and exceptional fecundity and reproductive potential^3,4^. *Physella acuta* has successfully invaded various natural and artificial freshwater habitats worldwide and is now found on all continents except Antarctica^1,2^.

From a biogeographical perspective, snail invasions have a significant negative effect on the environment, leading to the homogenization of fauna, extinction of vulnerable endemic species, and changes in biotic composition within invaded ecosystems^5,6^. The rapid displacement of native snails by *P. acuta* in several countries is a significant ecological concern^7,8^. Elevated population densities of *P. acuta* in new habitats affect native fauna and severely threaten plants of economic importance in greenhouses^8^. Furthermore, apart from its ecological implications, *P. acuta* has significant zoonotic medical relevance, as it can potentially act as a transitional carrier for several human trematode ailments, including echinostomiasis and fascioliasis^9,10^. *Physella acuta* has also been associated with outbreaks of cervical dermatitis in humans, serving as an intermediate host for *Trichobilharzia*, the causative agent of cervical dermatitis in many European countries^11^.

*Physella acuta* was first introduced in the northeastern province of Thailand in 2001 and subsequently spread to both the northern and southern provinces by 2005^12^. This snail is commonly found in various habitats, including drainage ditches, rivers, paddy fields, canals, and artificial habitats such as concrete pots or ponds^12,13^. Previous studies have indicated that *P. acuta* in Thailand serves as a host for *Trichodina unionis*, the causative agent of aquatic animal diseases. The disease adversely impacts both captive and wild fish and can negatively affect aquaculture and the economy^14^. Additionally, *P. acuta* has been documented to be infected with trematode cercariae, including xiphidiocercariae and echinostome cercariae. These indicate the potential of this invasive snail to act as an intermediate host for trematode cercariae in non-native regions^13,15^.

Molecular phylogeography and population genetic structure are powerful tools that can reveal crucial aspects of invasion processes, such as migration, gene flow, genetic drift, and population expansion^1,2^. The mitochondrial cytochrome c oxidase subunit I (*COI*) and 16S rDNA genes, along with other genes such as the internal transcribed spacer I (ITS1) region, nuclear 18S rRNA, and 28S rRNA genes are commonly used for genetic analysis in *P. acuta* snails^1,2,16–18^. However, mitochondrial markers are often better at detecting subtle genetic subdivisions owing to their reduced effective population size and lack of recombination^1,19^. Therefore, mitochondrial markers are often used to characterize population genetic structures and identify source populations^1,2^.

Efforts have been made to explain the population genetic structure of *P. acuta* on both regional^20,21^ and global scales^1,2^. Previous studies have revealed that the genetic diversity and phylogeography of *P. acuta* can be classified into two clades, A and B. Clade A exhibits a global distribution, whereas clade B is confined to the western United States^1^. However, sample sizes from some Asian countries have been too small to assess the population genetic structure, indicating that further investigation would be useful^1,21^. The *COI* gene has been used in previous studies to identify clades of *P. acuta* discovered in Thailand^1,21^, but the underlying population genetic structure and demographic history have never been explored. Therefore, the present study aimed to shed light on the phylogeography and genetics of *P. acuta* in Thailand in the context of previous research using mitochondrial and nuclear sequences. We aimed to provide broad insights into the genetic diversity, genetic structure, and demographic history of *P. acuta* in Thailand. These findings establish a foundation for understanding the genetic diversity of *P. acuta* and contribute to developing guidelines for controlling invasive snails in Thailand.

## Results

### Molecular identification of *P. acuta*

To identify *P. acuta*, PCR-based analysis and sequencing of the *COI* (509 bp), 16S rDNA (426 bp), and ITS1 (506–512 bp) genes were performed, together with a BLASTN search of the sequences. A total of 322 samples (161 samples for each gene) of *Physella* sp. were analyzed for mitochondrial *COI* and 16S rDNA genes. The nucleotide sequences generated in this study were submitted to GenBank under the accession numbers OR738467-OR738627 and OR738836-OR738996 for *COI* and 16S rDNA, respectively. The *COI* and 16S rDNA sequences in this study were mostly similar to *P. acuta* in the GenBank database, with the highest similarity of 99.41–100% and 99.52–100% for *COI* and 16S rDNA genes, respectively. Seventy-one random samples (GenBank accession numbers OR738997–OR739067) were analyzed using the ITS1 region and were identified as *P. acuta* with 97.34–100% identity after a BLASTN search.

### Phylogenetic and haplotype network analyses

**Figure 1:**
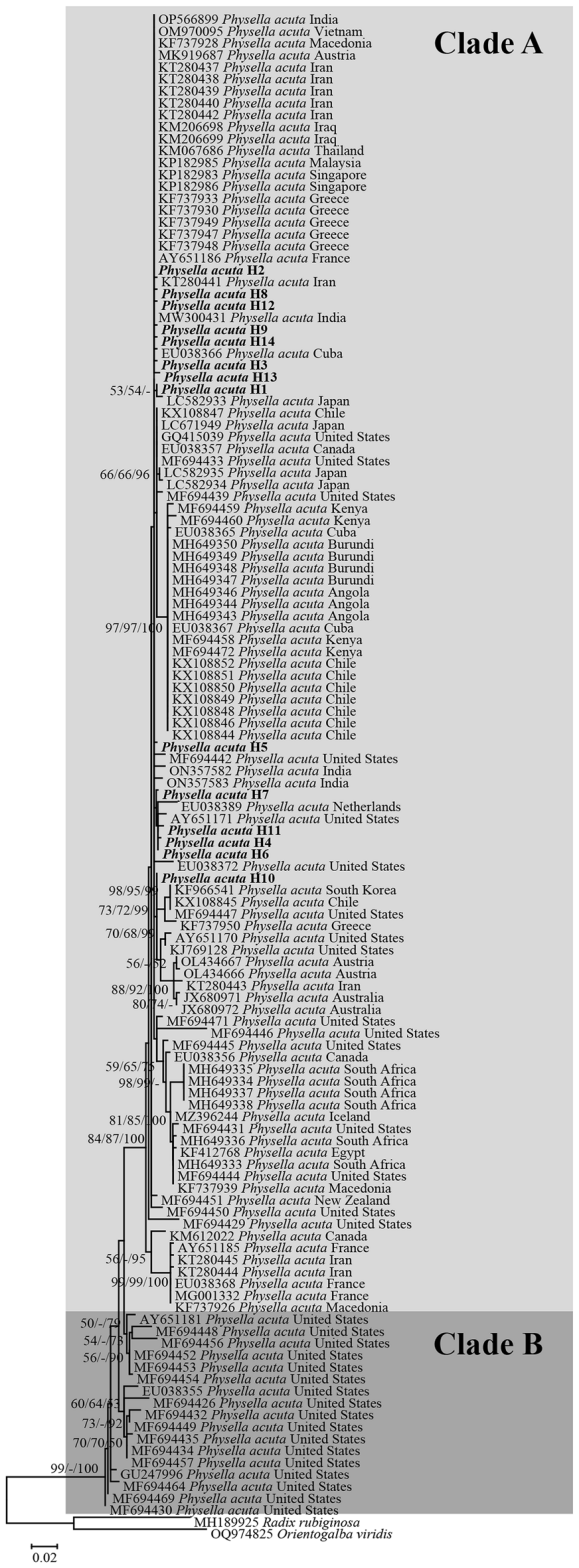
Maximum likelihood phylogenetic tree based on *COI* sequences (509 bp) of 14 *P. acuta* haplotypes from Thailand, along with an additional 112 sequences from various geographical regions. Support values (ML bootstrap/NJ bootstrap/Bayesian posterior probabilities) are shown at the branch points. A dash (-) on the branches indicates less than 50% support value or that a certain grouping was not seen by that analysis method. Bold letters indicate the sequences obtained in this study. *Radix rubiginosa* and *Orientogalba viridis* were utilized as outgroups.

**Figure 2:**
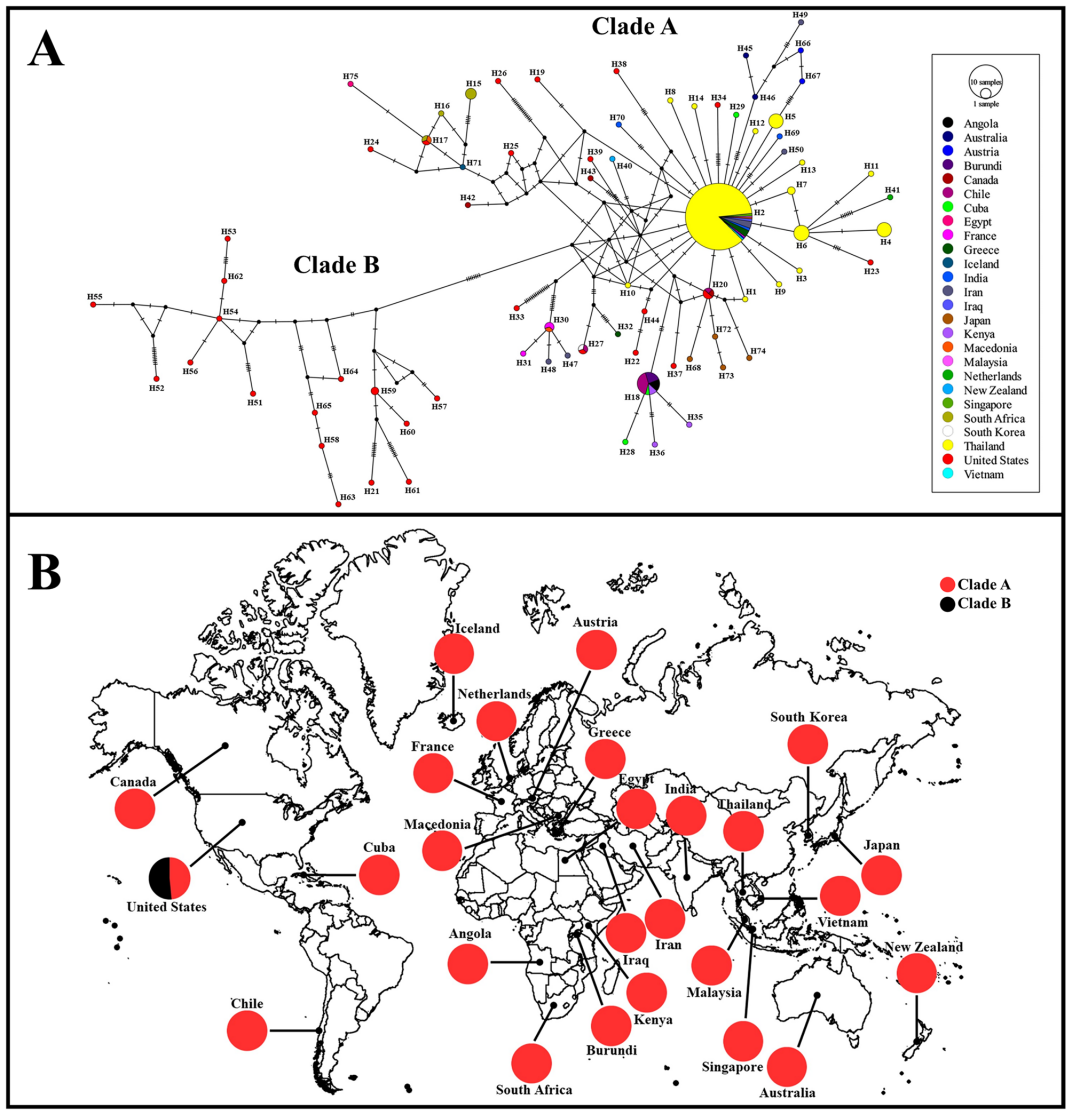
Haplotype network (**A**) and geographical distribution of clades (**B**) for *P. acuta* from 26 countries, based on *COI* sequences (509 bp). In the haplotype network, the size of the circles represents the proportion of samples found in each haplotype, with small black dots indicating hypothetical missing haplotypes. Each mutation between haplotypes is indicated by a bar.

**Figure 3:**
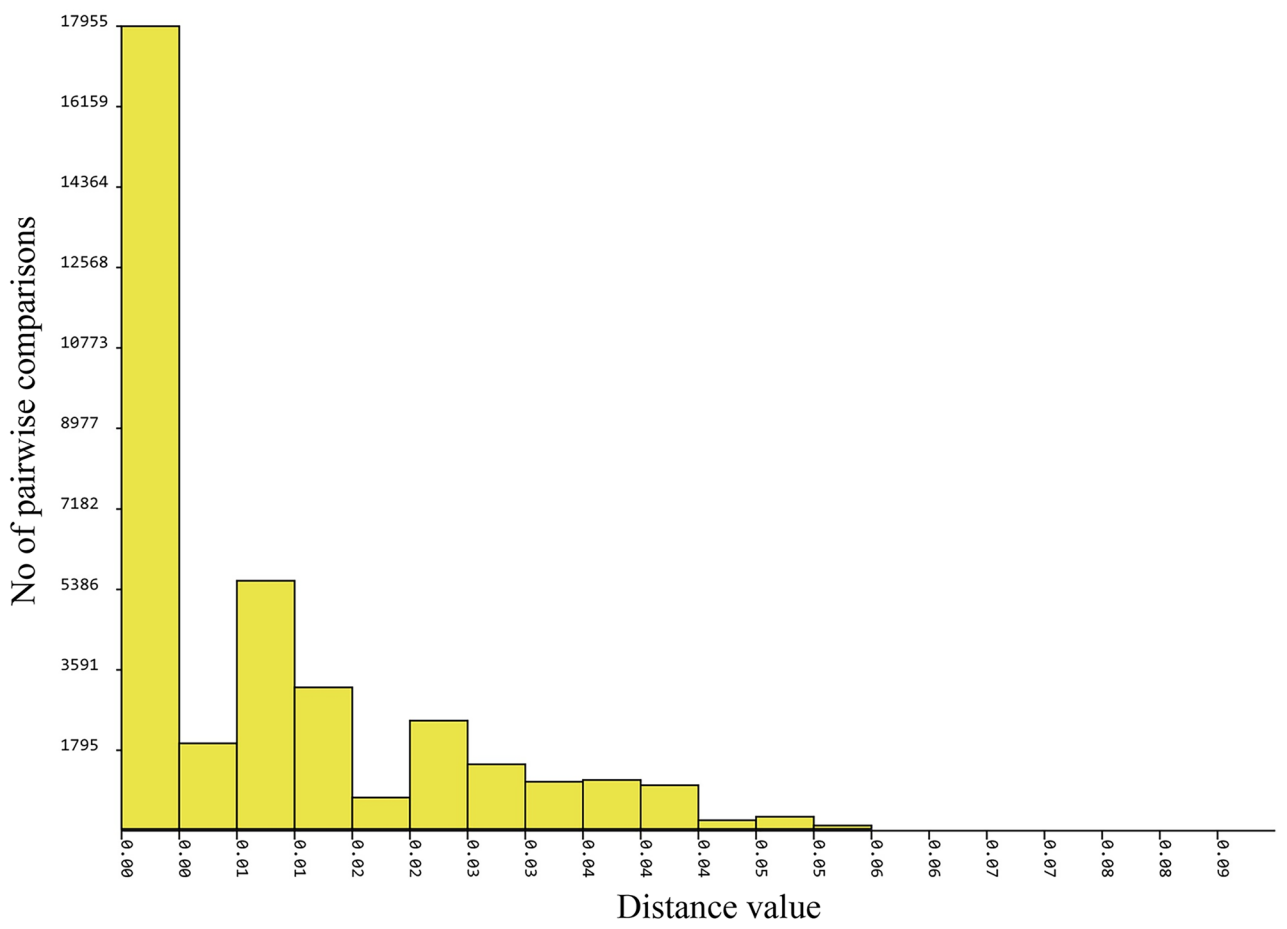
Results of the Automatic Barcode Gap Discovery (ABGD) analysis conducted on 161 COI sequences of *P. acuta*.

Based on a previous study on *P. acuta*, two distinct clades, known as Clade A and B, were identified. In the current phylogenetic tree and haplotype network analyses based on the *COI* gene, all our *P. acuta* sequences, representing 14 haplotypes, were grouped into clade A. Clade A was the largest group and the most widely distributed across various regions worldwide, whereas clade B was limited to the western United States (Figs. 1, 2). Genetic distances within clade A and clade B are 0.75% and 2.12%, respectively, while the genetic distance between clade A and B is 3.51%. Additionally, analysis using the Automatic Barcode Gap Discovery (ABGD) method for the COI marker revealed no evident barcode gap, as illustrated in Fig. 3.

**Figure 4:**
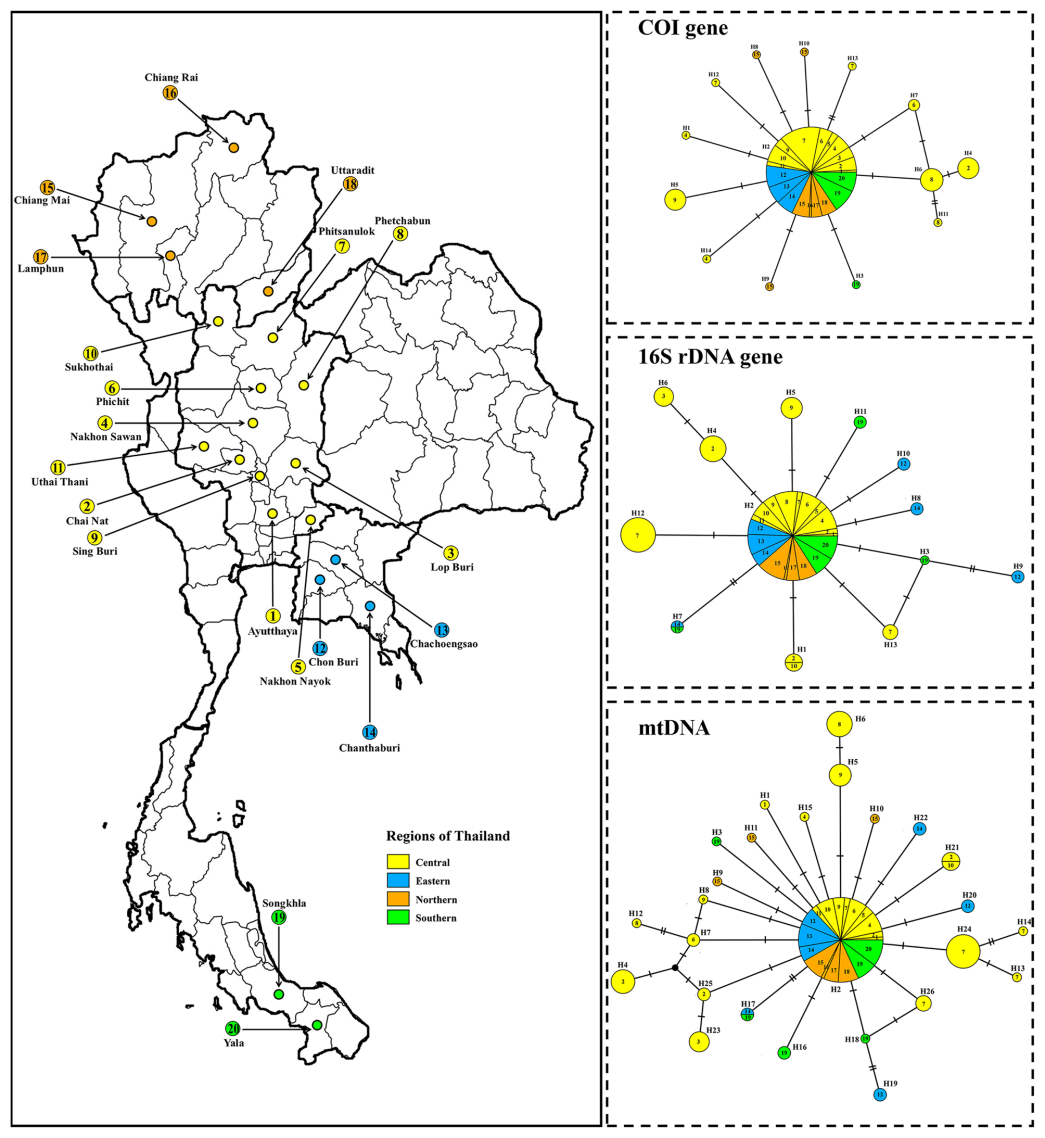
Map and haplotype network of *P. acuta* from 20 provinces in four geographical regions of Thailand based on *COI*, 16S rDNA, and mtDNA (COI + 16S rDNA) sequences. Each haplotype is represented by a circle, and circle sizes are proportional to haplotype frequency. Colors indicate the geographic origin of the haplotypes. The small black dots indicate hypothetical missing haplotypes. Each mutation between haplotypes is indicated by a bar. The number inside each circle indicates the provinces.

In the haplotype networks, we genetically analyzed 161 individual *P. acuta* samples from 20 populations in Thailand to examine their relationships with geographical locations within the country. Network analysis of *P. acuta* based on *COI* sequences (509 bp) revealed 14 distinct haplotypes (H1–H14) with 14 variable sites (Supplementary Table S1). Among these haplotypes, 13 (92.86%) were unique to specific populations, whereas one (7.14%) was shared by at least two localities. Furthermore, eight populations (40%) comprised multiple haplotypes, and 12 (60%) had a single haplotype. The average haplotype diversity (h) was high at 0.3674, while nucleotide diversity (π) was relatively low, with an average of 0.0010 (Table 1). The analysis of the 16S rDNA (161sequences) identified 13 variable nucleotide sites, which were identified and classified into 13 haplotypes (H1–H13) (Supplementary Table S1). Of these, 10 haplotypes (76.92%) were unique, and three (23.08%) were shared by multiple populations. Among the populations, seven (35%) consisted of multiple haplotypes, whereas 13 (65%) had a single haplotype. Average haplotype and nucleotide diversity values were 0.5498 and 0.0019, respectively (Table 1). Meanwhile, the MJ network of combined mtDNA sequences was classified into 26 haplotypes (H1–H26). Among these, 88.5% (23 haplotypes) were unique, and 11.54% (3 haplotypes) were shared by at least two populations. The estimates of haplotype and nucleotide diversity are shown in Table 1. Haplotype H2 was the most common haplotype for both the COI, 16S rDNA, and mtDNA, shared between populations from different regions (Fig. 4). Additionally, the haplotype network structure of *P. acuta* in Thailand formed a star-like structure, with the H2 haplotype serving as the prominent central haplotype surrounded by several low-frequency haplotypes (Fig. 4).

**Table 1:** Genetic diversity of *P. acuta* from 20 provinces in four regions of Thailand based on COI, 16S rDNA, and mtDNA sequences.

Regions	*COI*					
	N	S	H	Uh	Hd ± SD	π ± SD
Central	90	10	10	9	0.5253 ± 0.0606	0.0015 ± 0.0013
Ayutthaya	1	0	1	0	NA	NA
Chai Nat	13	2	2	1	0.5385 ± 0.0602	0.0021 ± 0.0017
Lop Buri	5	0	1	0	0.0000 ± 0.0000	0.0000 ± 0.0000
Nakhon Sawan	9	2	3	2	0.4167 ± 0.1907	0.0009 ± 0.0009
Nakhon Nayok	3	0	1	0	0.0000 ± 0.0000	0.0000 ± 0.0000
Phichit	8	1	2	1	0.4286 ± 0.1687	0.0008 ± 0.0009
Phitsanulok	21	3	3	2	0.1857 ± 0.1102	0.0006 ± 0.0007
Phetchabun	9	2	2	2	0.2222 ± 0.1662	0.0009 ± 0.0009
Sing Buri	11	1	2	1	0.5091 ± 0.1008	0.0010 ± 0.0010
Sukhothai	8	0	1	0	0.0000 ± 0.0000	0.0000 ± 0.0000
Uthai Thani	2	0	1	0	0.0000 ± 0.0000	0.0000 ± 0.0000
Eastern	26	0	1	0	0.0000 ± 0.0000	0.0000 ± 0.0000
Chon Buri	10	0	1	0	0.0000 ± 0.0000	0.0000 ± 0.0000
Chachoengsao	8	0	1	0	0.0000 ± 0.0000	0.0000 ± 0.0000
Chanthaburi	8	0	1	0	0.0000 ± 0.0000	0.0000 ± 0.0000
Northern	24	3	4	3	0.2391 ± 0.1129	0.0005 ± 0.0006
Chiang Mai	11	3	4	3	0.4909 ± 0.1754	0.0011 ± 0.0011
Chiang Rai	1	0	1	0	NA	NA
Lamphun	5	0	1	0	0.0000 ± 0.0000	0.0000 ± 0.0000
Uttaradit	7	0	1	0	0.0000 ± 0.0000	0.0000 ± 0.0000
Southern	21	1	2	1	0.0952 ± 0.0843	0.0002 ± 0.0004
Songkhla	12	1	2	1	0.1667 ± 0.1343	0.0003 ± 0.0005
Yala	9	0	1	0	0.0000 ± 0.0000	0.0000 ± 0.0000
Total	161	14	14	13	0.3674 ± 0.0489	0.0010 ± 0.0009

Regions	16S rDNA					
	N	S	H	Uh	Hd ± SD	π ± SD
Central	90	6	7	5	0.6826 ± 0.0446	0.0022 ± 0.0017
Ayutthaya	1	0	1	0	NA	NA
Chai Nat	13	2	3	1	0.5128 ± 0.1436	0.0018 ± 0.0016
Lop Buri	5	0	1	1	0.0000 ± 0.0000	0.0000 ± 0.0000
Nakhon Sawan	9	0	1	0	0.0000 ± 0.0000	0.0000 ± 0.0000
Nakhon Nayok	3	0	1	0	0.0000 ± 0.0000	0.0000 ± 0.0000
Phichit	8	0	1	0	0.0000 ± 0.0000	0.0000 ± 0.0000
Phitsanulok	21	2	3	2	0.4095 ± 0.1205	0.0015 ± 0.0014
Phetchabun	9	0	1	0	0.0000 ± 0.0000	0.0000 ± 0.0000
Sing Buri	11	1	2	1	0.5455 ± 0.0722	0.0013 ± 0.0013
Sukhothai	8	1	2	0	0.4286 ± 0.1687	0.0010 ± 0.0012
Uthai Thani	2	0	1	0	0.0000 ± 0.0000	0.0000 ± 0.0000
Eastern	26	7	5	3	0.4646 ± 0.1158	0.0021 ± 0.0017
Chon Buri	10	4	3	2	0.6222 ± 0.1383	0.0033 ± 0.0025
Chachoengsao	8	0	1	0	0.0000 ± 0.0000	0.0000 ± 0.0000
Chanthaburi	8	3	3	1	0.6071 ± 0.1640	0.0022 ± 0.0019
Northern	24	0	1	0	0.0000 ± 0.0000	0.0000 ± 0.0000
Chiang Mai	11	0	1	0	0.0000 ± 0.0000	0.0000 ± 0.0000
Chiang Rai	1	0	1	0	NA	NA
Lamphun	5	0	1	0	0.0000 ± 0.0000	0.0000 ± 0.0000
Uttaradit	7	0	1	0	0.0000 ± 0.0000	0.0000 ± 0.0000
Southern	21	4	4	2	0.3476 ± 0.1276	0.0011 ± 0.0011
Songkhla	12	4	4	2	0.5606 ± 0.1540	0.0019 ± 0.0016
Yala	9	0	1	0	0.0000 ± 0.0000	0.0000 ± 0.0000
Total	161	13	13	10	0.5498 ± 0.0459	0.0019 ± 0.0015

Regions	mtDNA (COI + 16S rDNA)					
	N	S	H	Uh	Hd ± SD	π ± SD
Central	90	16	16	14	0.8242 ± 0.0307	0.0018 ± 0.0012
Ayutthaya	1	0	1	0	NA	NA
Chai Nat	13	4	4	2	0.6923 ± 0.1154	0.0019 ± 0.0014
Lop Buri	5	0	1	1	0.0000 ± 0.0000	0.0000 ± 0.0000
Nakhon Sawan	9	2	3	2	0.4167 ± 0.1907	0.0005 ± 0.0005
Nakhon Nayok	3	0	1	0	0.0000 ± 0.0000	0.0000 ± 0.0000
Phichit	8	1	2	1	0.4286 ± 0.1687	0.0005 ± 0.0005
Phitsanulok	21	5	5	4	0.5476 ± 0.1188	0.0009 ± 0.0007
Phetchabun	9	2	2	2	0.2222 ± 0.1662	0.0005 ± 0.0005
Sing Buri	11	2	3	2	0.6182 ± 0.1038	0.0011 ± 0.0009
Sukhothai	8	1	2	0	0.4286 ± 0.1687	0.0005 ± 0.0005
Uthai Thani	2	0	1	0	0.0000 ± 0.0000	0.0000 ± 0.0000
Eastern	26	7	5	3	0.4646 ± 0.1158	0.0009 ± 0.0008
Chon Buri	10	4	3	2	0.6222 ± 0.1383	0.0015 ± 0.0011
Chachoengsao	8	0	1	0	0.0000 ± 0.0000	0.0000 ± 0.0000
Chanthaburi	8	3	3	1	0.6071 ± 0.1640	0.0009 ± 0.0008
Northern	24	3	4	3	0.2391 ± 0.1129	0.0002 ± 0.0004
Chiang Mai	11	3	4	3	0.4909 ± 0.1754	0.0006 ± 0.0006
Chiang Rai	1	0	1	0	NA	NA
Lamphun	5	0	1	0	0.0000 ± 0.0000	0.0000 ± 0.0000
Uttaradit	7	0	1	0	0.0000 ± 0.0000	0.0000 ± 0.0000
Southern	21	5	5	3	0.4238 ± 0.1305	0.0006 ± 0.0006
Songkhla	12	5	5	3	0.6667 ± 0.1409	0.0010 ± 0.0008
Yala	9	0	1	0	0.0000 ± 0.0000	0.0000 ± 0.0000
Total	161	27	26	23	0.6878 ± 0.0404	0.0014 ± 0.0009

N = number of *P. acuta* examined, S = number of polymorphic sites, H = number of haplotypes, Uh = unique haplotypes, Hd = haplotype diversity, π = nucleotide diversity, NA = not calculated because of small sample size.

To assess the relationship between *P. acuta* from Thailand and other countries, we constructed haplotype networks using 273 *COI* sequences. This dataset includes 161 sequences from the current study and 112 sequences obtained from GenBank. Among the 273 sequences, 75 haplotypes (H1–H75) were identified. The 161 sequences from Thailand used in the current study were represented by 14 haplotypes (H1–H14). In most *P. acuta* sequences from Thailand, 128 sequences (accounting for 79.50% of all samples in the present study) belonged to the H2 haplotype. Haplotype H2 included sequences from ten countries (in addition to Thailand) across Asia and Europe (Fig. 2).

### Cluster analysis

**Figure 5:**
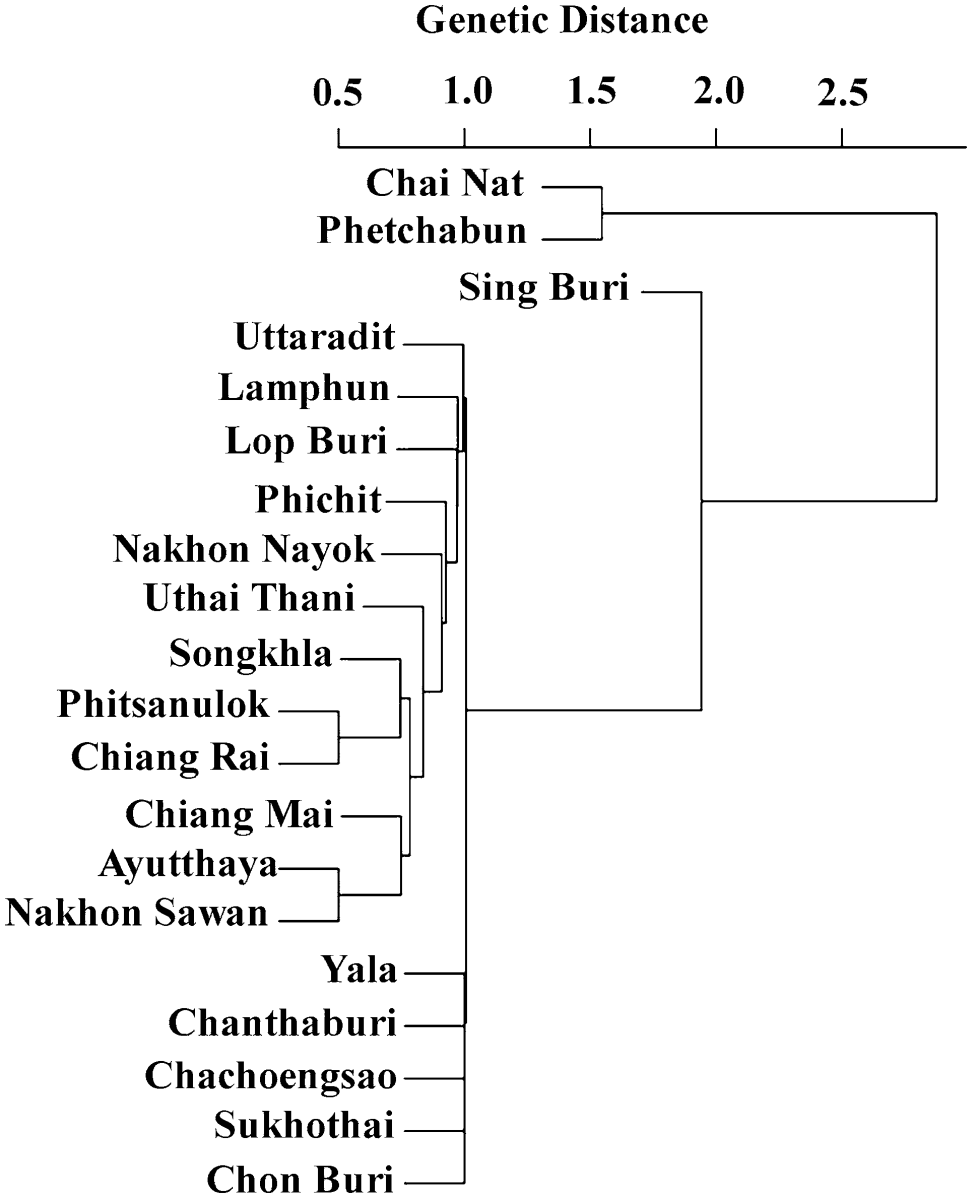
Cluster analysis of genetic relationships among 20 *P. acuta* populations in Thailand, based COI sequences.

Cluster analysis of *P. acuta* populations in Thailand revealed two major clusters. Cluster 1 comprised sample populations from Chai Nat and Phetchabun. Cluster 2 was subdivided into two sub-clusters. The first sub-cluster included the snail population from Sing Buri, while the second sub-cluster encompassed populations from Uttaradit, Lamphun, Lop Buri, Phichit, Nakhon Nayok, Uthai Thani, Songkhla, Phitsanulok, Chiang Rai, Chiang Mai, Ayutthaya, Nakhon Sawan, Yala, Chanthaburi, Chachoengsao, Sukhothai, and Chon Buri (Fig. 5).

### Genetic structure analysis

**Figure 6:**
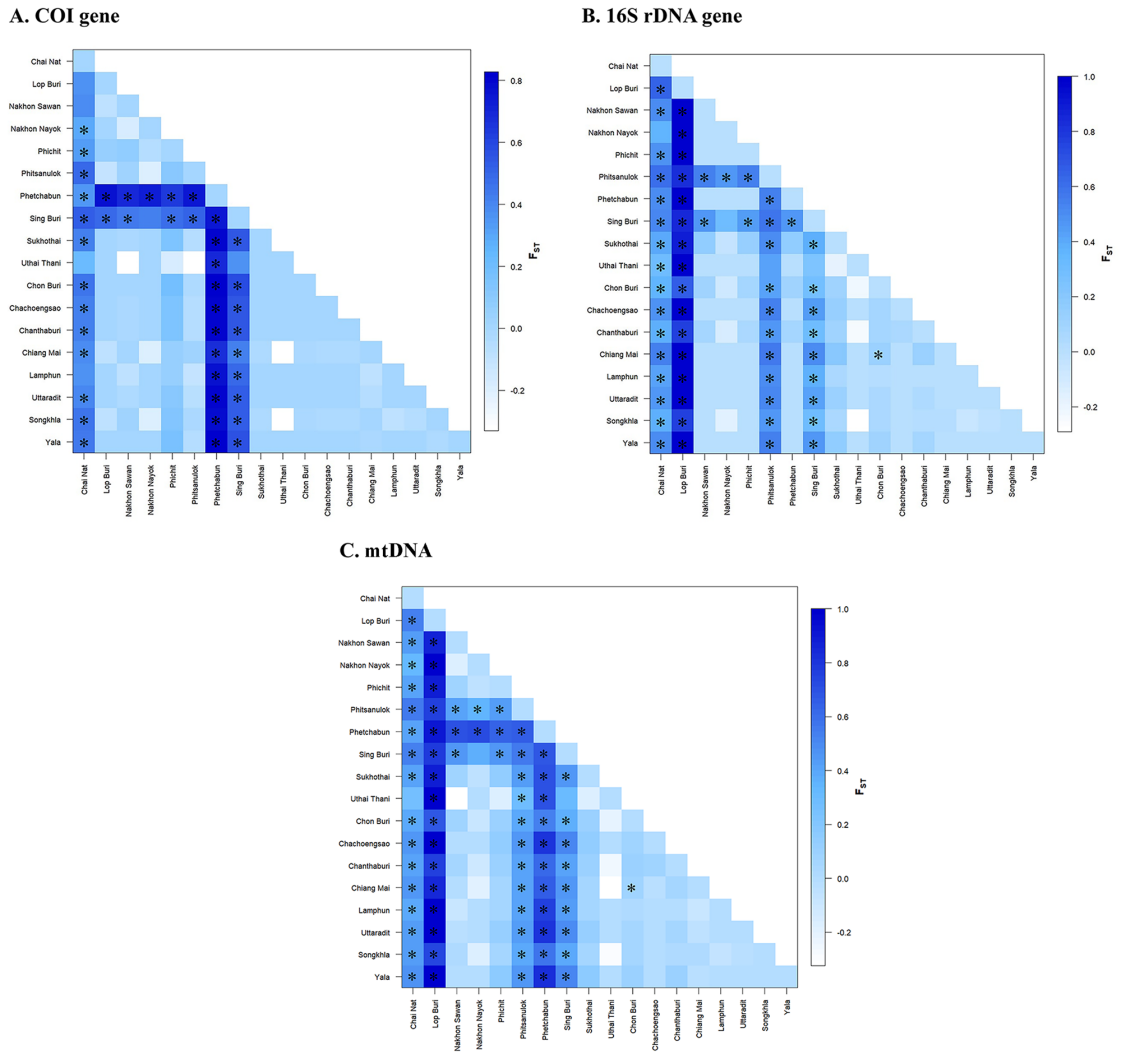
Graph of pairwise F_ST_ distance matrices between populations of *P. acuta* in Thailand based on *COI* (**A**),16S rDNA (**B**), and mtDNA (COI + 16S rDNA) (**C**) sequences. Darker blue indicates a higher pairwise F_ST_ value and lighter blue indicates a lower value. Asterisks (*) indicate F_ST_ values with statistically significant differences (*P* < 0.05).

**Figure 7:**
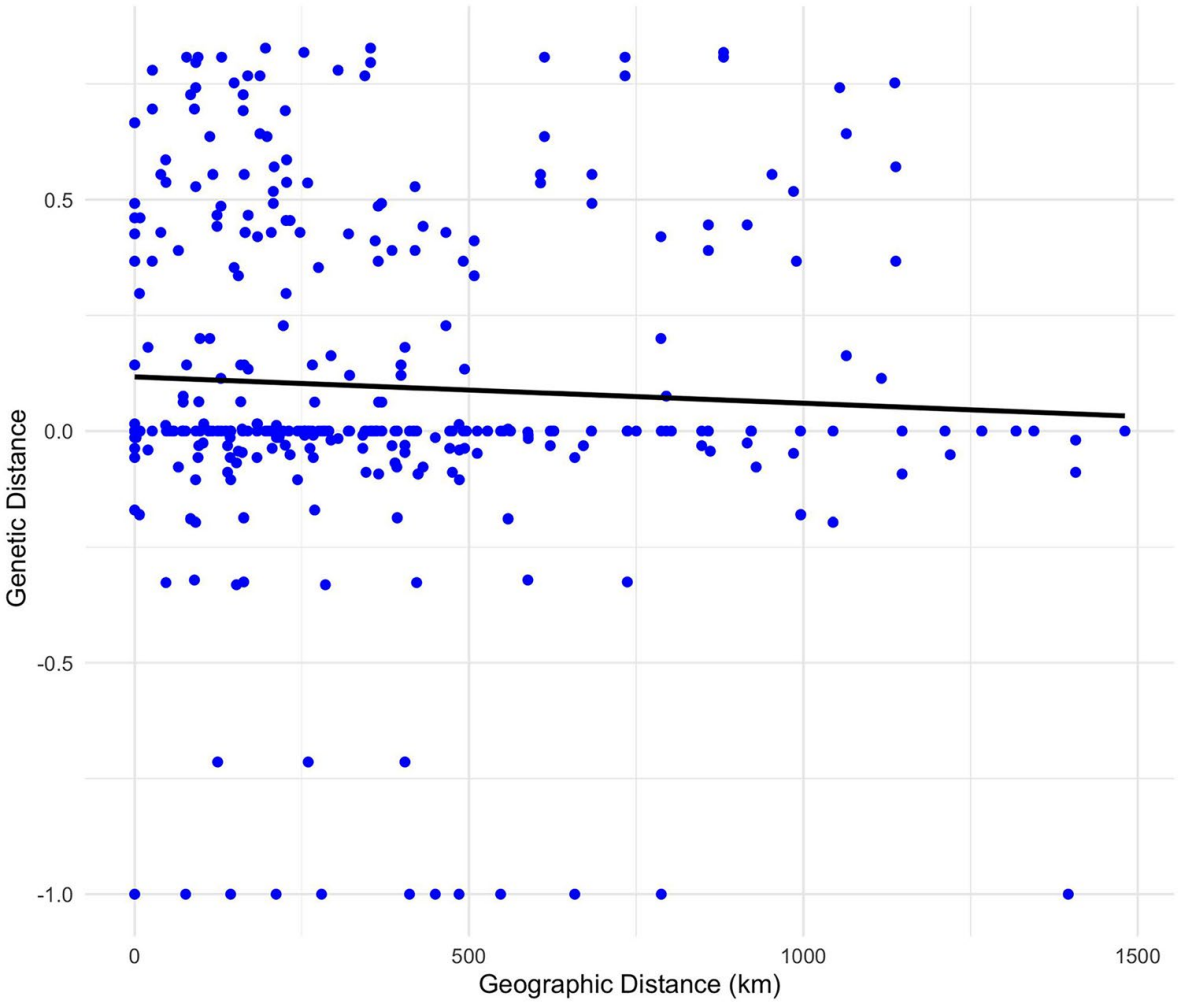
Correlation of geographic distance (in kilometers) and genetic distance among 161 individuals of 20 populations of *P. acuta* based on COI sequences.

Population pairwise F_ST_ analysis indicated varying degrees of genetic differentiation among *P. acuta* populations in the 18 provinces sampled in Thailand. Pairwise F_ST_ analysis of *P. acuta* based on *COI* sequences revealed that 72.55% (111 of 153) were not genetically significantly different. Similarly, analyses based on 16S rDNA and mtDNA revealed that 94 (61.44%) and 80 (52.29%) of the 153 pairwise comparisons, respectively, were not genetically significantly different. The provinces in the eastern, northern, and southern regions were not genetically significantly different from others in their regions. However, in the central region, some provinces displayed significant genetic differences from others within the same region (Fig. 6, Supplementary Table S2). Moreover, an analysis using a Mantel test to assess the relationship between geographical distances and genetic differentiation revealed non-significant findings (r = 0.0115, *p* = 0.338). Despite detecting a very weak positive correlation between geographic and genetic distances, the association was not statistically significant (R^2^ = 0.003, *p* = 0.299) (Fig. 7).

### Demographic history

**Figure 8:**
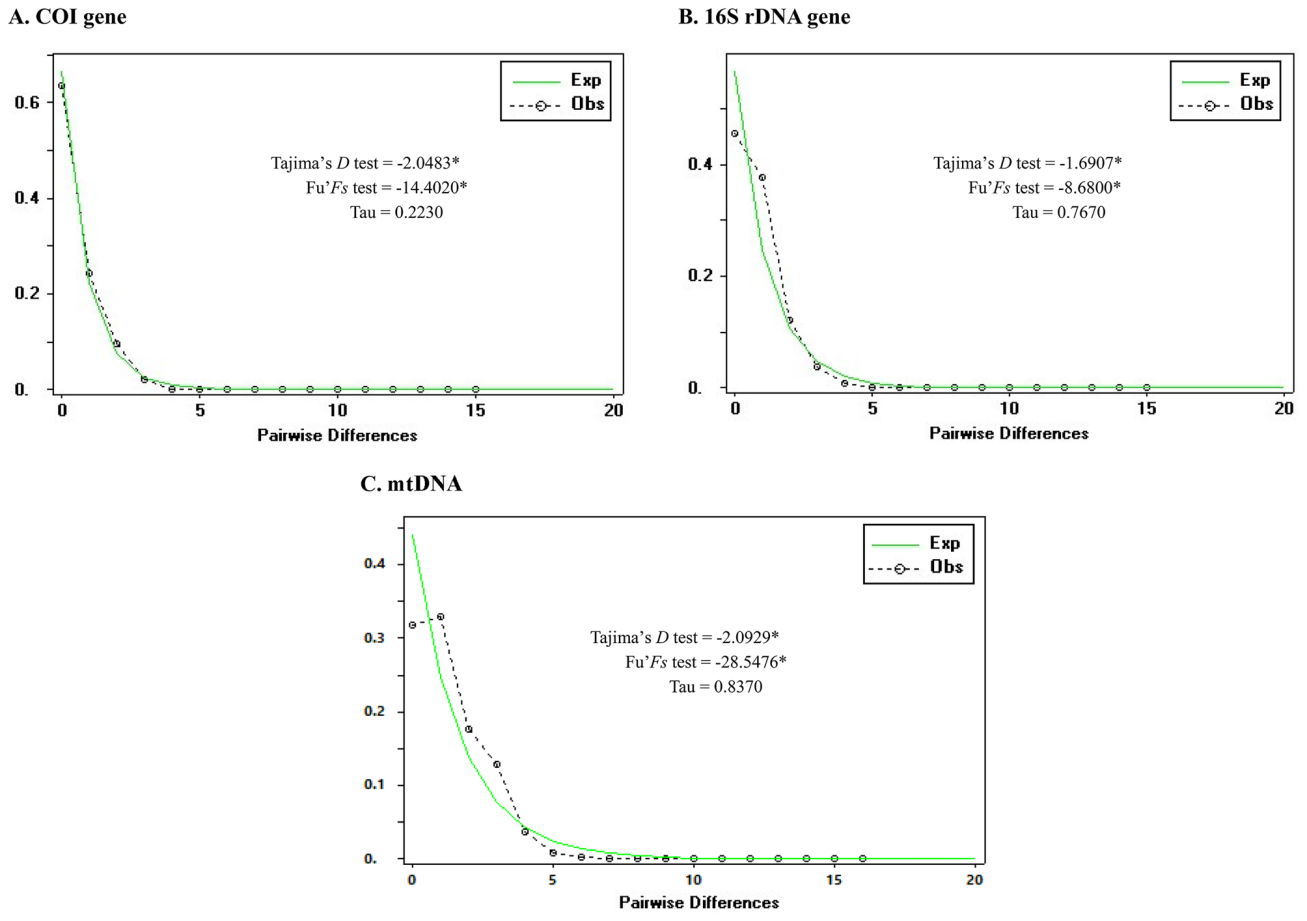
Mismatch distribution of *P. acuta* from Thailand based on *COI* (**A**), 16S rDNA (**B**), and mtDNA (COI + 16S rDNA) sequences. The black dotted lines with circles illustrate the observed frequency of pairwise differences, and the green lines represent the expected values under the sudden population expansion model. Asterisks indicate values levels of statistical significance (*P* < 0.05).

Mismatch distribution analysis of *P. acuta* based on *COI*,16S rDNA, and mtDNA sequences revealed a unimodal mismatch graph with non-significant values for the sum of squares deviation (SSD = 0.0001, *P* > 0.05 in *COI*; SSD = 0.0011, *P* > 0.05 in 16S rDNA; SSD = 0.0017, *P* > 0.05 in mtDNA) and Harpending’s raggedness index (HRI = 0.1819, *P* > 0.05 in *COI*; HRI = 0.0799, *P* > 0.05 in 16S rDNA; HRI = 0.0349, *P* > 0.05 in mtDNA). The results of the neutrality tests were highly significant negative values for both Tajima’s D and Fu’s Fs tests (Fig. 8).

## Discussion

In the present study, we identified the species and phylogenetic clades of *P. acuta* in many provinces of Thailand using mitochondrial markers (*COI* and 16S rDNA) and nuclear genetic markers (ITS1). We evaluated the phylogeography, genetic diversity, population structure, and demographic history of *P. acuta* in Thailand. Previous research has identified several clades of *P. acuta* worldwide, including Thailand^1,2,21^. Nevertheless, to the best of our knowledge, our data are the first to include 16S rDNA and ITS1 sequences reported from Thailand and provide additional insights into the haplotype distribution, population structure, and demographic history of *P. acuta* in Thailand.

Phylogenetic analysis of *P. acuta* from Thailand in the present study resulted in the classification of the species into clade A. This conclusion was further reinforced by the haplotype network analysis. Notably, clade A has been previously documented globally^1^. These data indicate that the *P. acuta* Thai strains are closely related to those from multiple countries. *Physella acuta*, which is native to North America^22^, was first recorded outside of its native range in the Bordeaux region of France in 1805, more than 200 years ago. It is believed to have been introduced via the cotton trade from Mississippi, USA, during the eighteenth century^1^. However, within less than a century, populations were reported across Eastern Europe, Africa, Asia, South America, the Middle East, the Caribbean, New Zealand, and Australia^1^. The invasion of the African continent occurred between the 1940s and 1950s, leading to reported populations in South Africa^23^, Nigeria^24^, and Namibia^25^. In Asia, *P. acuta* was recorded in Malaysia and Thailand in the 1970s^26^. Subsequently, during the 1980s, the species spread to numerous other countries in Asia, including Hong Kong, Singapore, and Australasia^1^. In Thailand, *P. acuta* was found to be more dispersed in the northeastern provinces in the 2001s, and in the northern and southern provinces in the 2005s^12^. Since then, *P. acuta* has been reported to have a widespread distribution across all regions of Thailand, as documented by Tantrawatpan et al.^21^. Previous studies have illustrated that *P. acuta* from different countries on many continents has a close evolutionary relationship^1,2^. Ebbs et al.^1^ studied the phylogeography and genetics of the globally invasive *P. acuta* and hypothesized that this snail in Western Europe (France and England) likely maintained its first invasive populations. The phenomenon of close evolutionary relationships among snails from different regions worldwide has frequently been observed, especially in invasive or alien species, such as *Pomacea canaliculata*^27^, *Indoplanorbis exustus*^28^, and *Achatina fulica*^29^.

In Thailand, we identified 14 *COI*, 13 16S rDNA, 26 mtDNA haplotypes in 161 *P. acuta* samples from 20 different populations. Our number of *P. acuta* haplotypes was relatively high compared to that in a previous study. Tantrawatpan et al.^21^ reported genetic variation in *P. acuta* in 12 provinces of Thailand based on the *COI* gene and identified seven different haplotypes. The variation in haplotype numbers between our study and a previous study^21^ may be due to the smaller sample size in the previous study in comparison to our current study, or it could be due to the high mutation rate of mitochondrial DNA^30^.

Our haplotype distribution results on *P. acuta* in Thailand revealed that most *P. acuta* haplotypes in Thailand were distinct haplotypes discovered in particular regions. In contrast, a mere fraction of the haplotypes were shared, accounting for only 7.14% (*COI*), 23.08% (16S rDNA), 11.54% (mtDNA) of the total samples analyzed. Haplotype H2 was the most common haplotype for both the *COI*,16S rDNA, and mtDNA shared between populations from different regions. When assessing the relationship between *P. acuta* from Thailand and those from other countries, we found that haplotype H2 was shared among *P. acuta* populations from 10 countries across Asia and Europe. Haplotype H2 is widespread and may be the ancestral haplotype of the *P. acuta* Thai strain. Our findings are in concordance with those of a previous study by Ebbs et al.^1^, who reported that the invasive haplotypes of *P. acuta* were found only in clade A. The presence of shared haplotypes observed in this study indicates gene flow or migration between distant populations^31^.

Genetic diversity indices of *P. acuta* from Thailand showed that the overall haplotype diversity was high, but nucleotide diversity was low. Higher values of haplotype diversity relative to nucleotide diversity indicate rapid expansion from the founding populations or continued expansion^32^. Additionally, this indicates the adaptation of snails to enable the colonization of diverse habitats^33^. This phenomenon has been previously observed in other invasive snails in Thailand, including *P. canaliculata* and *P. maculata*^27^.

In this study, we found that the population genetic structure of *P. acuta* in many provinces of Thailand showed that most comparisons were not genetically significantly different. This observation suggests that gene flow within the *P. acuta* population in Thailand may have led to genetic homogeneity^34^. However, in the central region, certain provinces displayed significant genetic distinctions from others within the same region. Furthermore, cluster analysis of *P. acuta* populations identified two major clusters: cluster 1 comprised Chai Nat and Phetchabun populations, while cluster 2 exhibited two sub-clusters. The first sub-cluster encompassed the Sing Buri population, while the second sub-cluster consisted of populations from multiple provinces. Numerous factors influence genetic structure and population distribution. An isolation-by-distance test revealed no significant correlation between genetic and geographical distances among *P. acuta* populations, indicating that gene flow is not restricted by distance. The genetic structure could be influenced by various factors such as habitat type, climate, the presence of predators, interspecific competition, and various other environmental factors in different habitats^35–37^.

The mismatch distribution analysis of *P. acuta* in Thailand revealed a unimodal mismatch graph with non-significant values for the sum-of-squares deviation and Harpending’s raggedness index. These results suggest sudden population expansion^38^. This expansion is consistent with the star-like structure observed in the median-joining network, with the most common haplotypes in the star’s center^39^. Additionally, population expansion was supported by highly significant negative values in both Tajima’s D and Fu’s Fs tests. Our findings suggested that *P. acuta* is invasive and is undergoing population expansion in Thailand. However, its limited dispersal ability, anthropogenic activity, hydrological connectivity, and flash floods have facilitated its expansion into new areas^20,22^. In recent years, human activities, such as water transport, shipment of exotic plants, aquarium trade, and industrial aquaculture, have significantly accelerated the dispersal of this snail^22^. In particular, in the ornamental trade, as reported by Ng et al.^12^, *P. acuta* was discovered on aquatic plants that were being transported for sale in an ornamental pet trade shop located in a market in Bangkok. Ornamental trade may have contributed significantly to their spread in Thailand. Moreover, *P. acuta* can disperse through river basins through water flow, particularly in flooded rice fields, allowing snails to move freely^20^. Bird-mediated natural dispersal mechanisms can effectively disperse snails over large distances and through natural barriers in various areas^40^. Life-cycle characteristics, such as high proliferation rates, a wide range of ecological tolerance, and rapid adaptation to new environments, are regarded as pivotal factors contributing to the successful dispersal of *P. acuta* into new areas^3,41^. *P. acuta* can self-fertilize and reproduce multiple times per year, coupled with rapid juvenile growth, leading to a rapid increase in its population and outcompeting other snails^42^. The capacity for self-fertilization would have enabled the rapid spread of the original haplotypes from the initial snail populations, as seen in the invasion from the United States to Africa^2^.

*Physella acuta* is known to displace native snails and become the dominant species over very short periods of time^7,23^. The geographical expansion of *P. acuta* may have important parasitological implications as it serves as a natural intermediate host for numerous trematodes, including *Fasciola hepatica*^10^, *Echinoparyphium* sp.^43^, *Echinostoma revolutum*^9^, and *Trichobilharzia physellae*^11^. Notably, *Trichobilharzia*, which belongs to the family Schistosomatidae and is also known as the avian schistosome, is a causative agent of avian cervical dermatitis worldwide^44^. The presence of the parasitosis vector in a geographical area facilitates the establishment of the parasite life cycle and increases the chances of rapid infection by secondary intermediate and definitive hosts^45^. Furthermore, *P. acuta* invasion accelerates the spread of parasites because of their high expansiveness, resulting in the occurrence of a parasitic life cycle in non-endemic areas^46^. Therefore, the invasion of *P. acuta* may be key to the successful spread of several parasites into new areas.

## Conclusions

In the present study, we confirmed that *P. acuta* from Thailand belongs to Clade A. Genetic diversity and population genetic structure analyses suggested gene flow within the *P. acuta* population in Thailand. Haplotype network analysis revealed a star-like structure with the most common haplotypes in the star’s center, a characteristic of the recent demographic expansion of populations. Population expansion was also supported by the unimodal graph of the mismatch distribution analysis and the highly negative values of the neutrality indices. Our findings suggested that *P. acuta* is invasive and is undergoing population expansion in Thailand. Understanding the invasion and levels of gene flow of *P. acuta* intermediate hosts within Thailand can be valuable not only for studying its genetic divergences but also for surveillance in areas vulnerable to the circulation of trematode parasites.

## Materials and methods

### Ethic and biosafety statement

The research involving invertebrate animals (snails) in this study received ethical approval from the Center for Animal Research at Naresuan University under Project Ethics Approval No. NU-AQ640803. The experimental protocols for biosafety and biosecurity were approved by the Naresuan University Institutional Biosafety Committee under Project Approval No: NUIBC MI 64-09-34.

### Snail collection and identification

*Physella acuta* snails were randomly collected from 25 distinct locations across 20 provinces in central, eastern, northern, and southern Thailand. Details of these locations are listed in Table 2. Snail samples were obtained from their natural habitats, including paddy fields, lotus ponds, and canals. The collection method involved handpicking and scooping. The snails were then transported at ambient temperature to the Department of Microbiology and Parasitology, Faculty of Medical Science, Naresuan University, Phitsanulok, Thailand. The snails were cleaned with tap water and identified using standard morphological criteria described by Paraense and Pointier^47^, Wethington et al.^48^, and Ng et al.^12^. After identification, the snail bodies were separated from their shells, and approximately 25 mg of foot tissue from each individual was excised and stored at − 20 °C for future DNA analysis.

**Table 2:** Sampling information of *Physella acuta* in this study.

Code	Province	District	Region	Habitat	Number of individuals	GenBank accession number		
						*COI*	16S rDNA	ITS1
AYA	Ayutthaya	Bang Pa-in	Central	Paddy field	1	OR738467	OR738836	–
CNT	Chai Nat	Manorom	Central	Paddy field	13	OR738468–OR738480	OR738837–OR738849	OR738997–OR739007
LRI	Lop Buri	Tha Wung	Central	Lotus pond	5	OR738481–OR738485	OR738850–OR738854	–
NSN1	Nakhon Sawan	Phayuha Khiri	Central	Canal	3	OR738486–OR738488	OR738855–OR738857	OR739008–OR739010
NSN2	Nakhon Sawan	Banphot Phisai	Central	Paddy field	6	OR738489–OR738494	OR738858–OR738863	OR739011–OR739015
NYK	Nakhon Nayok	Ban Na	Central	Lotus pond	3	OR738495–OR738497	OR738864–OR738866	–
PCT1	Phichit	Sam Ngam	Central	Paddy field	4	OR738498–OR738501	OR738867–OR738870	OR739016
PCT2	Phichit	Bueng Na Rang	Central	Lotus pond	4	OR738502–OR738505	OR738871–OR738874	OR739017, OR739018
PLK	Phitsanulok	Mueang Phitsanulok	Central	Paddy field	21	OR738506–OR738526	OR738875–OR738895	OR739019–OR739030
PNB	Phetchabun	Nong Phai	Central	Lotus pond	9	OR738527–OR738535	OR738896–OR738904	OR739031–OR739036
SBR	Sing Buri	In Buri	Central	Paddy field	11	OR738536–OR738546	OR738905–OR738915	OR739037, OR739038
STI1	Sukhothai	Si Nakhon	Central	Lotus pond	2	OR738547, OR738548	OR738916, OR738917	OR739039
STI2	Sukhothai	Mueang Sukhothai	Central	Paddy field	6	OR738549–OR738554	OR738918–OR738923	–
UTI	Uthai Thani	Mueang Uthai Thani	Central	Paddy field	2	OR738555, OR738556	OR738924, OR738925	OR739040
CBI	Chon Buri	Mueang Chon Buri	Eastern	Lotus pond	10	OR738557–OR738566	OR738926–OR738935	–
CCO1	Chachoengsao	Mueang Chachoengsao	Eastern	Paddy field	3	OR738567–OR738569	OR738936–OR738938	–
CCO2	Chachoengsao	Bang Nam Priao	Eastern	Paddy field	5	OR738570–OR738574	OR738939–OR738943	–
CTI	Chanthaburi	Laem Sing	Eastern	Lotus pond	8	OR738575–OR738582	OR738944–OR738951	–
CMI1	Chiang Mai	San Sai	Northern	Paddy field	4	OR738583–OR738586	OR738952–OR738955	OR739041–OR739044
CMI2	Chiang Mai	Mae Rim	Northern	Paddy field	7	OR738587–OR738593	OR738956–OR738962	OR739045–OR739051
CRI	Chiang Rai	Mae Lao	Northern	Paddy field	1	OR738594	OR738963	OR739052
LPN	Lamphun	Mae Tha	Northern	Lotus pond	5	OR738595–OR738599	OR738964–OR738968	OR739053–OR739057
UTT	Uttaradit	Tron	Northern	Paddy field	7	OR738600–OR738606	OR738969–OR738975	OR739058–OR739060
SKA	Songkhla	Mueang Songkhla	Southern	Lotus pond	12	OR738607–OR738618	OR738976–OR738987	–
YLA	Yala	Mueang Yala	Southern	Lotus pond	9	OR738619–OR738627	OR738988–OR738996	OR739061–OR739067
Total					161			

### DNA extraction, amplification, and DNA sequencing

Genomic DNA was extracted from individual *P. acuta* samples using the NucleoSpin® Tissue Kit (Macherey–Nagel, Duren, Germany), following the manufacturer’s instructions. The quality of the genomic DNA was assessed by electrophoresis on a 0.8% agarose gel in 1 × TBE buffer at 100 V for 35 min, and the DNA was stored at − 20 °C until further analysis. Polymerase chain reaction (PCR) was performed to amplify a portion of the mitochondrial *COI* and 16S rDNA genes and the ITS1 region using the primers specified in Table 3. The PCR reaction (total volume 30 µl) consisted of 15 µl of OnePCR Ultra (Bio-helix, New Taipei, Taiwan), 1.5 µl of each primer at 5 µM (0.25 µM), 9 µl of distilled water, and 3 µl of the DNA template (20–200 ng). PCR was performed using a Biometra TOne Thermal Cycler (Analytik Jena AG, Jena, Germany), as shown in Table 3. The PCR products were purified using a NucleoSpin® Gel and PCR Clean-Up Kit (Macherey–Nagel, Düren, Germany) according to the manufacturer’s instructions. Purified PCR products were analyzed by 1.2% agarose gel electrophoresis at 100 V, stained with ethidium bromide, destained with distilled water, and visualized and photographed under UV light. Purified PCR products were sequenced by Macrogen Inc. (Seoul, Korea).

**Table 3:** Details of primers used in this study.

Gene or region	Primer	Amplicon size (bp)	Thermal profile	References
*COI*	LCO1490_forward 5′-GGTCAACAAATCATAAAGATATTGG-3′ HCO2198_reverse 5′-TAAACTTCAGGGTGACCAAAAAATCA-3′	710	95 °C for 5 min 1 cycle, 34 cycles of 95 °C for 30 s, 49 °C for 90 s, and 72 °C for 30 s, and 72 °C for 10 min 1 cycle	Folmer et al.^49^
16S rDNA	16Sar_forward 5′-CGCCTGTTTATCAAAAACAT-3′ 16Sbr_reverse 5′-CCGGTCTGAACTCAGATCACGT-3′	500		Kessing et al.^50^
ITS1	ITS1-S_forward 5′-CCATGAACGAGGAATTCCCAG-3′ 5.8S-AS_reverse 5′-TTAGCAAACCGACCCTCAGAC-3′	802	94 °C for 10 min 1 cycle, 25 cycles of 94 °C for 30 s, 53 °C for 60 s, and 72 °C for 1 min, and 72 °C for 7 min 1 cycle	Ebbs et al.^1^

### Phylogenetic and haplotype analyses

The *COI*, 16S rDNA, and ITS1 genes were manually reviewed and edited using SeqMan II (DNASTAR, Madison, WI, USA). Nucleotide sequences were aligned and trimmed using the ClustalW algorithm in MEGA (version 7.0 program)^51^. All sequences obtained in this study were BLASTed against GenBank (http://blast.ncbi.nlm.nih.gov/Blast.cgi) for species identification. A phylogenetic tree was constructed using the maximum likelihood (ML), neighbor-joining (NJ), and Bayesian inference (BI) methods. ML phylogenetics was estimated using the Tamura 3-parameter model^52^, whereas the NJ tree was estimated using the Kimura 2-parameter model^53^ with bootstrap support of 1000 resamplings using MEGA version 7.0. BI analysis was conducted using the MrBayes 3.2.0 program^54^. Bayesian posterior probabilities (BPPs) were estimated via Markov chain Monte Carlo (MCMC) analysis with 2,000,000 generations. Trees were sampled every 1000th generation, resulting in 2000 trees. Additionally, genetic species delimitation was conducted using mtDNA COI sequences through the Automatic Barcode Gap Discovery (ABGD) method^55^. The online version of ABGD (http://wwwabi.snv.jussieu.fr/public/abgd/abgdweb.html) was employed with default settings and the K2P distance model.

To assess the relationship between *P. acuta* haplotypes from different geographic areas in Thailand and across the world, a median-joining network was constructed using PopART v1.7^56^. For the *COI* sequences, we generated a phylogeny and two median-joining networks to assess the genetic relationships among *P. acuta* populations within Thailand and compared them to populations in other countries. The grouping classifications in our phylogenetic tree and haplotype network were the same as those used by Ebbs et al.^1^.

### Genetic diversity, population genetic structure, and demographic history

Genetic diversity parameters, such as the number of segregation sites, number of haplotypes, haplotype diversity (h), and nucleotide diversity (π), were calculated using DnaSP version 5^57^ and Arlequin version 3.5.1.2^58^. Pairwise population F_ST_ calculations were performed using Arlequin software. To mitigate the influence of limited sample size, populations consisting of only one sample were excluded from the F_ST_ analysis. Neutrality indices (Fu’s Fs and Tajima’s D) were used to assess the hypothesis of selective neutrality using Arlequin. Mismatch distribution analysis was performed using the DnaSP and Arlequin programs to evaluate demographic equilibrium and examine sudden population expansion. The sum of squares deviation (SSD) and Harpending’s raggedness index^59^ were employed to assess the deviation from sudden or spatial expansion models using the Arlequin program. Cluster analysis was conducted using R software to explore the genetic structure of *P. acuta* populations in Thailand.

To assess the genetic relationships between the *COI* haplotypes of *P. acuta* in Thailand and those from other geographic regions, we added 112 sequences obtained from GenBank from outside Thailand. Genetic relationships between *COI* haplotypes were established using the median-joining (MJ) network algorithm^60^ in PopART v1.7.

### Association between geographical distance and genetic divergence

To evaluate whether geographical distance between populations explained genetic differentiation, we examined the relationship between genetic divergence and geographic distances (isolation by distance, IBD) with a Mantel test in software R using the package Vegan^61^. Additionally, we generated scatter plots to examine the relationships between individual pairwise genetic and geographical distances.

### Supplementary Information

Supplementary Information 1.

Supplementary Information 2.

## Data Availability

The sequences analysed during the current study are available in the GenBank with accession numbers. OR738467–OR738627 (*COI*), OR738836–OR738996 (16S rDNA), and OR738997–OR739067 (ITS1). The authors confirm that the data supporting the findings of this study are available within the article.
