# Supplementary material for: Genetic diversity and population structure of Physella acuta (Gastropoda: Physidae) in Thailand using mitochondrial gene markers: COI and 16S rDNA
Source: Sci Rep. 2024 Jun 7;14:13161. doi: 10.1038/s41598-024-64184-4 (PMC11161527; doi:10.1038/s41598-024-64184-4)
Supplement: Supplementary file 1 — Supplementary Information 1. [file 41598_2024_64184_MOESM1_ESM.docx]

**Supplementary Table S1.** List of the haplotypes identified in the *P. acuta* samples from Thailand based on COI, 16S rDNA, and mtDNA analyses.

| **Sample code** | **Haplotype** | | |
| --- | --- | --- | --- |
|  | **COI** | **16S rDNA** | **mtDNA** |
| P1733AYA_TH | H2 | H2 | H2 |
| P758CNT_TH | H4 | H4 | H4 |
| P759CNT_TH | H4 | H4 | H4 |
| P760CNT_TH | H2 | H4 | H25 |
| P761CNT_TH | H4 | H4 | H4 |
| P762CNT_TH | H4 | H4 | H4 |
| P763CNT_TH | H2 | H2 | H2 |
| P859CNT_TH | H2 | H1 | H21 |
| P860CNT_TH | H2 | H2 | H2 |
| P861CNT_TH | H4 | H4 | H4 |
| P862CNT_TH | H4 | H4 | H4 |
| P863CNT_TH | H2 | H1 | H21 |
| P1309CNT_TH | H2 | H4 | H25 |
| P1310CNT_TH | H4 | H4 | H4 |
| P2004LRI_TH | H2 | H6 | H23 |
| P2005LRI_TH | H2 | H6 | H23 |
| P2006LRI_TH | H2 | H6 | H23 |
| P2007LRI_TH | H2 | H6 | H23 |
| P2008LRI_TH | H2 | H6 | H23 |
| P1350NSN1_TH | H2 | H2 | H2 |
| P1351NSN1_TH | H2 | H2 | H2 |
| P1352NSN1_TH | H2 | H2 | H2 |
| P1361NSN2_TH | H14 | H2 | H15 |
| P1362NSN2_TH | H2 | H2 | H2 |
| P1363NSN2_TH | H1 | H2 | H1 |
| P1364NSN2_TH | H2 | H2 | H2 |
| P1365NSN2_TH | H2 | H2 | H2 |
| P1366NSN2_TH | H2 | H2 | H2 |
| P1941NYK_TH | H2 | H2 | H2 |
| P1944NYK_TH | H2 | H2 | H2 |
| P1945NYK_TH | H2 | H2 | H2 |
| P447PCT1_TH | H7 | H2 | H7 |
| P449PCT1_TH | H2 | H2 | H2 |
| P450PCT1_TH | H7 | H2 | H7 |
| P451PCT1_TH | H2 | H2 | H2 |
| P717PCT2_TH | H2 | H2 | H2 |
| P718PCT2_TH | H2 | H2 | H2 |
| P719PCT2_TH | H2 | H2 | H2 |
| P724PCT2_TH | H2 | H2 | H2 |
| P2PLK_TH | H2 | H13 | H26 |
| P3PLK_TH | H2 | H2 | H2 |
| P4PLK_TH | H2 | H13 | H26 |
| P27PLK_TH | H2 | H12 | H24 |

**Supplementary Table S1.** (continued)

| **Sample code** | **Haplotype** | | |
| --- | --- | --- | --- |
|  | **COI** | **16S rDNA** | **mtDNA** |
| P32PLK_TH | H12 | H12 | H13 |
| P46PLK_TH | H2 | H12 | H24 |
| P47PLK_TH | H2 | H12 | H24 |
| P73PLK_TH | H2 | H12 | H24 |
| P74PLK_TH | H2 | H12 | H24 |
| P75PLK_TH | H2 | H12 | H24 |
| P78PLK_TH | H2 | H12 | H24 |
| P98PLK_TH | H2 | H12 | H24 |
| P178PLK_TH | H2 | H12 | H24 |
| P179PLK_TH | H2 | H12 | H24 |
| P193PLK_TH | H2 | H13 | H26 |
| P287PLK_TH | H2 | H12 | H24 |
| P288PLK_TH | H13 | H12 | H14 |
| P289PLK_TH | H2 | H12 | H24 |
| P299PLK_TH | H2 | H12 | H24 |
| P300PLK_TH | H2 | H2 | H2 |
| P301PLK_TH | H2 | H12 | H24 |
| P399PNB_TH | H6 | H2 | H6 |
| P401PNB_TH | H6 | H2 | H6 |
| P402PNB_TH | H6 | H2 | H6 |
| P403PNB_TH | H6 | H2 | H6 |
| P410PNB_TH | H6 | H2 | H6 |
| P417PNB_TH | H6 | H2 | H6 |
| P418PNB_TH | H11 | H2 | H12 |
| P421PNB_TH | H6 | H2 | H6 |
| P422PNB_TH | H6 | H2 | H6 |
| P1120SBR_TH | H5 | H5 | H5 |
| P1121SBR_TH | H5 | H5 | H5 |
| P1122SBR_TH | H5 | H5 | H5 |
| P1123SBR_TH | H5 | H5 | H5 |
| P1124SBR_TH | H5 | H5 | H5 |
| P1125SBR_TH | H5 | H2 | H8 |
| P1126SBR_TH | H5 | H5 | H5 |
| P2022SBR_TH | H2 | H2 | H2 |
| P2023SBR_TH | H2 | H2 | H2 |
| P2025SBR_TH | H2 | H2 | H2 |
| P2036SBR_TH | H2 | H2 | H2 |
| P397STI1_TH | H2 | H2 | H2 |
| P398STI1_TH | H2 | H1 | H21 |
| P1584STI2_TH | H2 | H2 | H2 |
| P1589STI2_TH | H2 | H2 | H2 |
| P1591STI2_TH | H2 | H2 | H2 |
| P1601STI2_TH | H2 | H2 | H2 |
| P1607STI2_TH | H2 | H2 | H2 |
| P1609STI2_TH | H2 | H1 | H21 |

**Supplementary Table S1.** (continued)

| **Sample code** | **Haplotype** | | |
| --- | --- | --- | --- |
|  | **COI** | **16S rDNA** | **mtDNA** |
| P1321UTI_TH | H2 | H2 | H2 |
| P1322UTI_TH | H2 | H2 | H2 |
| P1734CBI_TH | H2 | H10 | H20 |
| P1735CBI_TH | H2 | H2 | H2 |
| P1736CBI_TH | H2 | H2 | H2 |
| P1737CBI_TH | H2 | H2 | H2 |
| P1738CBI_TH | H2 | H2 | H2 |
| P1739CBI_TH | H2 | H10 | H20 |
| P1740CBI_TH | H2 | H9 | H19 |
| P1741CBI_TH | H2 | H2 | H2 |
| P1742CBI_TH | H2 | H2 | H2 |
| P1743CBI_TH | H2 | H9 | H19 |
| P1761CCO1_TH | H2 | H2 | H2 |
| P1762CCO1_TH | H2 | H2 | H2 |
| P1763CCO1_TH | H2 | H2 | H2 |
| P1875CCO2_TH | H2 | H2 | H2 |
| P1876CCO2_TH | H2 | H2 | H2 |
| P1877CCO2_TH | H2 | H2 | H2 |
| P1878CCO2_TH | H2 | H2 | H2 |
| P1879CCO2_TH | H2 | H2 | H2 |
| P1668CTI_TH | H2 | H2 | H2 |
| P1669CTI_TH | H2 | H8 | H22 |
| P1670CTI_TH | H2 | H7 | H17 |
| P1671CTI_TH | H2 | H2 | H2 |
| P1673CTI_TH | H2 | H8 | H22 |
| P1674CTI_TH | H2 | H2 | H2 |
| P1675CTI_TH | H2 | H2 | H2 |
| P1677CTI_TH | H2 | H2 | H2 |
| P475CMI1_TH | H10 | H2 | H11 |
| P476CMI1_TH | H9 | H2 | H10 |
| P477CMI1_TH | H2 | H2 | H2 |
| P478CMI1_TH | H2 | H2 | H2 |
| P519CMI2_TH | H2 | H2 | H2 |
| P525CMI2_TH | H2 | H2 | H2 |
| P526CMI2_TH | H2 | H2 | H2 |
| P532CMI2_TH | H2 | H2 | H2 |
| P535CMI2_TH | H8 | H2 | H9 |
| P537CMI2_TH | H2 | H2 | H2 |
| P538CMI2_TH | H2 | H2 | H2 |
| P573CRI_TH | H2 | H2 | H2 |
| P305LPN_TH | H2 | H2 | H2 |
| P307LPN_TH | H2 | H2 | H2 |
| P311LPN_TH | H2 | H2 | H2 |
| P317LPN_TH | H2 | H2 | H2 |
| P323LPN_TH | H2 | H2 | H2 |

**Supplementary Table S1.** (continued)

| **Sample code** | **Haplotype** | | |
| --- | --- | --- | --- |
|  | **COI** | **16S rDNA** | **mtDNA** |
| P642UTT_TH | H2 | H2 | H2 |
| P643UTT_TH | H2 | H2 | H2 |
| P644UTT_TH | H2 | H2 | H2 |
| P649UTT_TH | H2 | H2 | H2 |
| P651UTT_TH | H2 | H2 | H2 |
| P654UTT_TH | H2 | H2 | H2 |
| P660UTT_TH | H2 | H2 | H2 |
| P1559SKA_TH | H2 | H2 | H2 |
| P1561SKA_TH | H2 | H11 | H16 |
| P1562SKA_TH | H2 | H2 | H2 |
| P1564SKA_TH | H2 | H3 | H18 |
| P1565SKA_TH | H2 | H7 | H17 |
| P1566SKA_TH | H2 | H2 | H2 |
| P1567SKA_TH | H3 | H2 | H3 |
| P1570SKA_TH | H2 | H11 | H16 |
| P1571SKA_TH | H2 | H2 | H2 |
| P1572SKA_TH | H2 | H2 | H2 |
| P1573SKA_TH | H2 | H2 | H2 |
| P1574SKA_TH | H2 | H2 | H2 |
| P342YLA_TH | H2 | H2 | H2 |
| P343YLA_TH | H2 | H2 | H2 |
| P344YLA_TH | H2 | H2 | H2 |
| P345YLA_TH | H2 | H2 | H2 |
| P346YLA_TH | H2 | H2 | H2 |
| P347YLA_TH | H2 | H2 | H2 |
| P348YLA_TH | H2 | H2 | H2 |
| P352YLA_TH | H2 | H2 | H2 |
| P360YLA_TH | H2 | H2 | H2 |
