# Supplementary material for: Genetic diversity and population structure of Physella acuta (Gastropoda: Physidae) in Thailand using mitochondrial gene markers: COI and 16S rDNA
Source: Sci Rep. 2024 Jun 7;14:13161. doi: 10.1038/s41598-024-64184-4 (PMC11161527; doi:10.1038/s41598-024-64184-4)
Supplement: Supplementary file 2 — Supplementary Information 2. [file 41598_2024_64184_MOESM2_ESM.docx]

**Supplementary Table S2.** Population pairwise F_ST_ between 18 populations of *P. acuta* based on COI, 16S rDNA, and mtDNA sequences.

| **Gene** | **Population** | **Chai Nat** | **Lop Buri** | **Nakhon Sawan** | **Nakhon Nayok** | **Phichit** | **Phitsanulok** | **Phetchabun** | **Sing Buri** | **Sukhothai** | **Uthai Thani** | **Chon Buri** | **Chachoengsao** | **Chanthaburi** | **Chiang Mai** | **Lamphun** | **Uttaradit** | **Songkhla** | **Yala** |
| --- | --- | --- | --- | --- | --- | --- | --- | --- | --- | --- | --- | --- | --- | --- | --- | --- | --- | --- | --- |
| COI | Chai Nat | - |  |  |  |  |  |  |  |  |  |  |  |  |  |  |  |  |  |
|  | Lop Buri | 0.367 | - |  |  |  |  |  |  |  |  |  |  |  |  |  |  |  |  |
|  | Nakhon Sawan | 0.390***** | -0.078 | - |  |  |  |  |  |  |  |  |  |  |  |  |  |  |  |
|  | Nakhon Nayok | 0.297 | 0.000 | -0.180 | - |  |  |  |  |  |  |  |  |  |  |  |  |  |  |
|  | Phichit | 0.336***** | 0.063 | 0.075 | -0.044 | - |  |  |  |  |  |  |  |  |  |  |  |  |  |
|  | Phitsanulok | 0.486***** | -0.105 | 0.016 | -0.197 | 0.114 | - |  |  |  |  |  |  |  |  |  |  |  |  |
|  | Phetchabun | 0.353***** | 0.768***** | 0.692***** | 0.727***** | 0.643***** | 0.752***** | - |  |  |  |  |  |  |  |  |  |  |  |
|  | Sing Buri | 0.528***** | 0.492***** | 0.442***** | 0.426 | 0.467***** | 0.518***** | 0.742***** | - |  |  |  |  |  |  |  |  |  |  |
|  | Sukhothai | 0.429***** | 0.000 | -0.014 | 0.000 | 0.143 | -0.057 | 0.808***** | 0.554***** | - |  |  |  |  |  |  |  |  |  |
|  | Uthai Thani | 0.228 | 0.000 | -0.321 | 0.000 | -0.171 | -0.331 | 0.696***** | 0.367 | 0.000 | - |  |  |  |  |  |  |  |  |
|  | Chon Buri | 0.461***** | 0.000 | 0.012 | 0.000 | 0.181 | -0.041 | 0.827***** | 0.586***** | 0.000 | 0.000 | - |  |  |  |  |  |  |  |
|  | Chachoengsao | 0.429***** | 0.000 | -0.014 | 0.000 | 0.143 | -0.057 | 0.808***** | 0.554***** | 0.000 | 0.000 | 0.000 | - |  |  |  |  |  |  |
|  | Chanthaburi | 0.429***** | 0.000 | -0.014 | 0.000 | 0.143 | -0.057 | 0.808***** | 0.554***** | 0.000 | 0.000 | 0.000 | 0.000 | - |  |  |  |  |  |
|  | Chiang Mai | 0.390***** | -0.089 | -0.002 | -0.187 | 0.063 | 0.015 | 0.666***** | 0.420***** | -0.032 | -0.325 | -0.009 | -0.032 | -0.032 | - |  |  |  |  |
|  | Lamphun | 0.367 | 0.000 | -0.078 | 0.000 | 0.063 | -0.105 | 0.768***** | 0.492***** | 0.000 | 0.000 | 0.000 | 0.000 | 0.000 | -0.089 | - |  |  |  |
|  | Uttaradit | 0.411***** | 0.000 | -0.031 | 0.000 | 0.120 | -0.069 | 0.796***** | 0.536***** | 0.000 | 0.000 | 0.000 | 0.000 | 0.000 | -0.046 | 0.000 | - |  |  |
|  | Songkhla | 0.455***** | -0.093 | 0.014 | -0.189 | 0.134 | -0.009 | 0.779***** | 0.538***** | -0.037 | -0.327 | -0.016 | -0.037 | -0.037 | 0.004 | -0.093 | -0.051 | - |  |
|  | Yala | 0.446***** | 0.000 | 0.000 | 0.000 | 0.163 | -0.048 | 0.818***** | 0.571***** | 0.000 | 0.000 | 0.000 | 0.000 | 0.000 | -0.019 | 0.000 | 0.000 | -0.026 | - |
| 16S rDNA | Chai Nat | - |  |  |  |  |  |  |  |  |  |  |  |  |  |  |  |  |  |
|  | Lop Buri | 0.651* | - |  |  |  |  |  |  |  |  |  |  |  |  |  |  |  |  |
|  | Nakhon Sawan | 0.508* | 1.000* | - |  |  |  |  |  |  |  |  |  |  |  |  |  |  |  |
|  | Nakhon Nayok | 0.369 | 1.000* | 0.000 | - |  |  |  |  |  |  |  |  |  |  |  |  |  |  |
|  | Phichit | 0.492* | 1.000* | 0.000 | 0.000 | - |  |  |  |  |  |  |  |  |  |  |  |  |  |
|  | Phitsanulok | 0.610* | 0.827* | 0.553* | 0.458* | 0.542* | - |  |  |  |  |  |  |  |  |  |  |  |  |
|  | Phetchabun | 0.508* | 1.000* | 0.000 | 0.000 | 0.000 | 0.553* | - |  |  |  |  |  |  |  |  |  |  |  |
|  | Sing Buri | 0.533* | 0.852* | 0.468* | 0.307 | 0.450* | 0.585* | 0.468* | - |  |  |  |  |  |  |  |  |  |  |
|  | Sukhothai | 0.404* | 0.881* | 0.163 | -0.044 | 0.143 | 0.512* | 0.163 | 0.381* | - |  |  |  |  |  |  |  |  |  |
|  | Uthai Thani | 0.308* | 1.000* | 0.000 | 0.000 | 0.000 | 0.417 | 0.000 | 0.234 | -0.171 | - |  |  |  |  |  |  |  |  |
|  | Chon Buri | 0.356* | 0.672* | 0.097 | -0.086 | 0.081 | 0.431* | 0.097 | 0.276* | 0.101 | -0.212 | - |  |  |  |  |  |  |  |
|  | Chachoengsao | 0.492* | 1.000* | 0.000 | 0.000 | 0.000 | 0.542* | 0.000 | 0.450* | 0.143 | 0.000 | 0.081 | - |  |  |  |  |  |  |
|  | Chanthaburi | 0.388* | 0.772* | 0.089 | -0.110 | 0.071 | 0.470* | 0.089 | 0.309* | 0.095 | -0.246 | 0.089 | 0.071 | - |  |  |  |  |  |
|  | Chiang Mai | 0.536* | 1.000* | 0.000 | 0.000 | 0.000 | 0.573* | 0.000 | 0.500* | 0.198 | 0.000 | 0.124* | 0.000 | 0.121 | - |  |  |  |  |
|  | Lamphun | 0.433* | 1.000* | 0.000 | 0.000 | 0.000 | 0.502* | 0.000 | 0.382* | 0.063 | 0.000 | 0.012 | 0.000 | -0.004 | 0.000 | - |  |  |  |
|  | Uttaradit | 0.475* | 1.000* | 0.000 | 0.000 | 0.000 | 0.530* | 0.000 | 0.431* | 0.120 | 0.000 | 0.062 | 0.000 | 0.051 | 0.000 | 0.000 | - |  |  |
|  | Songkhla | 0.388* | 0.769* | 0.007 | -0.159 | -0.005 | 0.459* | 0.007 | 0.297* | 0.061 | -0.291 | 0.066 | -0.005 | 0.014 | 0.028 | -0.063 | -0.019 | - |  |
|  | Yala | 0.508* | 1.000* | 0.000 | 0.000 | 0.000 | 0.553* | 0.000 | 0.468* | 0.163 | 0.000 | 0.097 | 0.000 | 0.089 | 0.000 | 0.000 | 0.000 | 0.007 | - |
| Concatenated mtDNA | Chai Nat | - |  |  |  |  |  |  |  |  |  |  |  |  |  |  |  |  |  |
|  | Lop Buri | 0.525* | - |  |  |  |  |  |  |  |  |  |  |  |  |  |  |  |  |
|  | Nakhon Sawan | 0.437* | 0.869* | - |  |  |  |  |  |  |  |  |  |  |  |  |  |  |  |
|  | Nakhon Nayok | 0.328* | 1.000* | -0.180 | - |  |  |  |  |  |  |  |  |  |  |  |  |  |  |
|  | Phichit | 0.401* | 0.880* | 0.075 | -0.043 | - |  |  |  |  |  |  |  |  |  |  |  |  |  |
|  | Phitsanulok | 0.561* | 0.765* | 0.420* | 0.347* | 0.430* | - |  |  |  |  |  |  |  |  |  |  |  |  |
|  | Phetchabun | 0.417* | 0.909* | 0.692* | 0.726* | 0.642* | 0.666* | - |  |  |  |  |  |  |  |  |  |  |  |
|  | Sing Buri | 0.530* | 0.774* | 0.452* | 0.369 | 0.460* | 0.562* | 0.677* | - |  |  |  |  |  |  |  |  |  |  |
|  | Sukhothai | 0.417* | 0.880* | 0.075 | -0.043 | 0.142 | 0.430* | 0.703* | 0.460* | - |  |  |  |  |  |  |  |  |  |
|  | Uthai Thani | 0.262 | 1.000* | -0.321 | 0.000 | -0.170 | 0.294* | 0.695* | 0.304 | -0.170 | - |  |  |  |  |  |  |  |  |
|  | Chon Buri | 0.399* | 0.671* | 0.079 | -0.085 | 0.101 | 0.379* | 0.529* | 0.377* | 0.101 | -0.212 | - |  |  |  |  |  |  |  |
|  | Chachoengsao | 0.456* | 1.000* | -0.014 | 0.000 | 0.142 | 0.444* | 0.807* | 0.506* | 0.142 | 0.000 | 0.080 | - |  |  |  |  |  |  |
|  | Chanthaburi | 0.407* | 0.771* | 0.055 | -0.110 | 0.095 | 0.401* | 0.607* | 0.406* | 0.095 | -0.245 | 0.089 | 0.071 | - |  |  |  |  |  |
|  | Chiang Mai | 0.447* | 0.834* | -0.002 | -0.187 | 0.063 | 0.419* | 0.666* | 0.450* | 0.063 | -0.325 | 0.088* | -0.031 | 0.057 | - |  |  |  |  |
|  | Lamphun | 0.395* | 1.000* | -0.077 | 0.000 | 0.062 | 0.399* | 0.767* | 0.440* | 0.062 | 0.000 | 0.012 | 0.000 | -0.003 | -0.089 | - |  |  |  |
|  | Uttaradit | 0.438* | 1.000* | -0.030 | 0.000 | 0.120 | 0.431* | 0.796* | 0.486* | 0.120 | 0.000 | 0.061 | 0.000 | 0.050 | -0.046 | 0.000 | - |  |  |
|  | Songkhla | 0.420* | 0.733* | 0.009 | -0.163 | 0.048 | 0.385* | 0.573* | 0.398* | 0.048 | -0.297 | 0.059 | -0.010 | 0.008 | 0.017 | -0.067 | -0.025 | - |  |
|  | Yala | 0.472* | 1.000* | 0.000 | 0.000 | 0.162 | 0.456* | 0.818* | 0.523* | 0.162 | 0.000 | 0.096 | 0.000 | 0.089 | -0.019 | 0.000 | 0.000 | 0.001 | - |
